# Pulmonary Vascular Resistance Estimated by Echocardiography in Dogs With Myxomatous Mitral Valve Disease and Pulmonary Hypertension Probability

**DOI:** 10.3389/fvets.2021.771726

**Published:** 2021-10-26

**Authors:** Ryohei Suzuki, Yunosuke Yuchi, Haruka Kanno, Takahiro Saito, Takahiro Teshima, Hirotaka Matsumoto, Hidekazu Koyama

**Affiliations:** Laboratory of Veterinary Internal Medicine, Faculty of Veterinary Medicine, School of Veterinary Science, Nippon Veterinary and Life Science University, Musashino, Japan

**Keywords:** canine, combined post- and pre-capillary pulmonary hypertension, congestive heart failure, post-capillary pulmonary hypertension, pulmonary arterial pressure, pulmonary vascular remodeling, right ventricular adaptation, tricuspid regurgitation

## Abstract

Post-capillary pulmonary hypertension (PH) is a life-threatening complication in dogs with myxomatous mitral valve disease (MMVD). An increase in pulmonary vascular resistance (PVR) is associated with post-capillary PH progression. In humans, PVR estimated by echocardiography (PVRecho) enables the non-invasive assessment of PVR in patients with PH. This study aimed to evaluate the clinical utility of PVRecho in dogs with MMVD, PH probability, and right-sided congestive heart failure (R-CHF). Dogs with MMVD and detectable tricuspid valve regurgitation were included in the study. Dogs were classified into three PH probability groups (low/intermediate/high) and according to the presence or absence of R-CHF. All dogs underwent echocardiographic measurements for right ventricular (RV) morphology and function. PVRecho was calculated by two methods using tricuspid valve regurgitation velocity and velocity–time integral of the pulmonary artery flow (PVRecho and PVRecho2). RV size indicators were significantly higher with a higher probability of PH. RV strain and velocity–time integral of the pulmonary artery flow in the high probability group were significantly lower than those in the other groups. Tricuspid valve regurgitation velocity, PVRecho, and PVRecho2 were significantly higher with an increase in PH probability. Logistic regression analysis revealed a significant association between the presence of R-CHF and increased PVRecho2 and end-diastolic RV internal dimension normalized by body weight. PVRecho and PVRecho2 showed significant differences among the PH probability groups. These non-invasive variables may be useful for the diagnosis and stratification of PH and the determination of the presence of R-CHF in dogs with MMVD.

## Introduction

Pulmonary hypertension (PH) is a life-threatening disease in dogs and is characterized by an increase in pulmonary arterial pressure (PAP) and/or pulmonary vascular resistance (PVR) (1, 2). PH is induced by various diseases in dogs, including pulmonary arterial disease, left heart disease, respiratory disease, hypoxia, pulmonary embolic disease, parasitic disease, or a combination of these (1). In particular, PH secondary to left heart disease is called post-capillary PH because the main factor of increasing PAP is considered to be the increased pulmonary venous pressure; it is classified into two subtypes: isolated post-capillary PH (Ipc-PH) and combined post- and pre-capillary PH (Cpc-PH) (1, 3–5). The former is caused solely by pulmonary venous congestion due to increasing left atrial pressure, whereas the latter is caused by the increase in PVR associated with pulmonary vascular remodeling in addition to the pathophysiology of Ipc-PH. As a result, the PAP in Cpc-PH was higher than that in Ipc-PH. Recent human studies have reported that Cpc-PH is significantly associated with poor prognosis in patients with left heart disease and heart failure (6–8). Therefore, PVR assessment is essential to determine the pathophysiology and prognosis of dogs with post-capillary PH.

The gold standard for determining the disease state of PH is by right heart catheterization (1, 9). Additionally, the absolute evaluation of PVR requires a catheterization-derived cardiac output and transpulmonary pressure gradient. However, the availability of right heart catheterization is limited, and its clinical use is restricted due to the need for anesthesia. Therefore, echocardiography has been used as an alternative to invasive indicators for disease evaluation. Specifically, in humans, PVR is estimated by echocardiography (PVRecho), which is calculated using tricuspid valve regurgitation (TR) velocity and the velocity–time integral of the pulmonary artery flow (PV VTI), has been reported to identify patients with elevated PVR (10–13). However, to the best of our knowledge, only one study has assessed the clinical utility of PVRecho in veterinary medicine (14).

The primary objective of this study was to evaluate the clinical utility of PVRecho in dogs with myxomatous mitral valve disease (MMVD), the most common cardiac disease in dogs (15). We hypothesized that PVRecho might provide additional information for the diagnosis and pathophysiological evaluation of PH in dogs with MMVD.

## Materials and Methods

This was a prospective observational study. Client-owned dogs that underwent cardiac screening at the Nippon Veterinary and Life Science University Veterinary Medical Teaching Hospital were recruited from October 2017 to May 2019. All procedures followed the Guidelines for Institutional Laboratory Animal Care and Use of Nippon Veterinary and Life Science University in Japan, and the study was approved by the Ethical Committee for Animal Use of the Nippon Veterinary and Life Science University Veterinary Medical Teaching Hospital, Japan (approval number: R2-5). Written informed consent authorizing the participation of the dogs in this study was obtained from all dog owners.

### Animals

Client-owned dogs with MMVD and detectable TR were prospectively included in our study. All dogs underwent complete physical examination, electrocardiography, blood pressure measurement by oscillometric method, and radiographic and echocardiographic examinations. Dogs were diagnosed as having MMVD based on echocardiographic findings of mitral valve thickening, prolapse, and mitral regurgitation (16, 17). Clinical diagnosis of TR was performed by inspecting the tricuspid valve from multiple views and using color Doppler echocardiography, and the peak TR velocity was obtained using the continuous-wave spectral Doppler method. Dogs that met the following criteria were excluded from the study: other cardiac diseases, diseases that might increase the PAP, such as pulmonary disease, thromboembolic disease, and neoplastic disease; diseases that might affect cardiac function, such as endocrine disease and systemic hypertension (systolic blood pressure ≥160 mmHg) (18), and/or missing data.

### Classification

Dogs with MMVD were divided into three groups (Stage B1, B2, and C/D) based on the MMVD severity noted by the American College of Veterinary Internal Medicine (ACVIM) consensus (15). Additionally, dogs were classified according to the PH probability noted on the ACVIM consensus using echocardiographic findings of the TR velocity and anatomical abnormalities of the right heart, pulmonary artery, and caudal vena cava into low, intermediate, and high probability groups (1). Furthermore, dogs with MMVD were classified by the presence or absence of right-sided congestive heart failure (R-CHF). Dogs were diagnosed as having R-CHF if they had ultrasonographic and/or radiographic findings indicative of ascites, pleural effusion, or pericardial effusion without any abnormalities other than PH that may have been responsible (17).

### Echocardiographic Evaluation of Right Heart

Conventional, two-dimensional, and Doppler echocardiographic examinations were performed using an echocardiographic system (Vivid E95, GE Healthcare, Tokyo, Japan) and a 3.5–6.9 MHz transducer by a single investigator (RS). Lead II electrocardiography was recorded simultaneously, and the results are displayed on the images. Non-sedated dogs that were manually restrained in right and left lateral recumbency. All data were obtained from at least five consecutive cardiac cycles in sinus rhythm. All images were analyzed by a single observer (YY) who was well trained by a cardiologist using an offline workstation (EchoPAC PC, Version 204, GE Healthcare, Tokyo, Japan).

All echocardiographic variables were measured using five consecutive cardiac cycles in sinus rhythm from high-quality images. To evaluate the right ventricular (RV) morphology, end-diastolic RV internal dimension (RVIDd) and end-diastolic and end-systolic RV area (RVEDA and RVESA, respectively) were measured using the left apical four-chamber view optimized for the right heart (RV focus view) (17, 19–22). RVIDd was measured as the largest diameter at the middle RV, parallel to the tricuspid annulus, using the B-mode method (17). RVEDA and RVESA were measured by tracing the endocardial border of the RV inflow region at end-diastole and end-systole, excluding the papillary muscles. These variables were normalized by body weight using the following formulas (21):


(1) $$\begin{array}{l} \text{RVIDd index}=\frac{\text{RVIDd }\left(\text{mm}\right)}{{\left[\text{body }weight\;\left(\text{kg}\right)\right]}^{0.327}} \end{array}$$

(2) $$\begin{array}{l} \text{RVEDA index}=\frac{\text{RVEDA }\left({\text{cm}}^{2}\right)}{{\left[\text{body weight }\left(\text{kg}\right)\right]}^{0.624}} \end{array}$$

(3) $$\begin{array}{l} \text{RVESA index}=\frac{\text{RVEDA }\left({\text{cm}}^{2}\right)}{{\left[\text{body weight }\left(\text{kg}\right)\right]}^{0.628}} \end{array}$$

Pulmonary artery to aortic diameter ratio (PA/Ao) was also obtained using the right parasternal short-axis view at the level of the heart base, as described previously (23).

For the RV functional assessment, tricuspid annular plane systolic excursion (TAPSE), RV functional area change (RV FAC), tissue Doppler imaging-derived peak systolic myocardial velocity of the lateral tricuspid annulus (RV s'), RV myocardial performance index (RV MPI), and PV VTI were measured as described previously (21–24). All RV functional indices, except PV VTI, were obtained using the RV focus view. The TAPSE was measured using the B-mode method, as described previously (17, 25). The TAPSE and RV FAC were normalized by body weight using the following formulas (25, 26):


(4) $$\begin{array}{l} \text{TAPSEn=}\frac{\left(\text{TAPSE }[\text{cm}]\right)}{{\left(\text{body weight }[\text{kg}]\right)}^{\text{0}.\text{284}}} \end{array}$$

(5) $$\begin{array}{l} \text{RV FACn=}\frac{\left(\text{RV FAC }[\%]\right)}{{\left(\text{body weight }[\text{kg}]\right)}^{-\text{0}.\text{097}}} \end{array}$$

RV MPI was obtained from the tissue Doppler imaging-derived lateral tricuspid annular motion wave, and calculated by dividing the sum of isovolumic contraction and relaxation time by ejection time. Isovolumic contraction and relaxation time were calculated by subtracting the interval from the end of the late-diastolic tricuspid annular motion wave to the onset of the early-diastolic tricuspid annular motion wave by the duration of the systolic tricuspid annular motion wave. Ejection time was defined as the duration of the systolic tricuspid annular motion wave (22).

**Figure 1:**
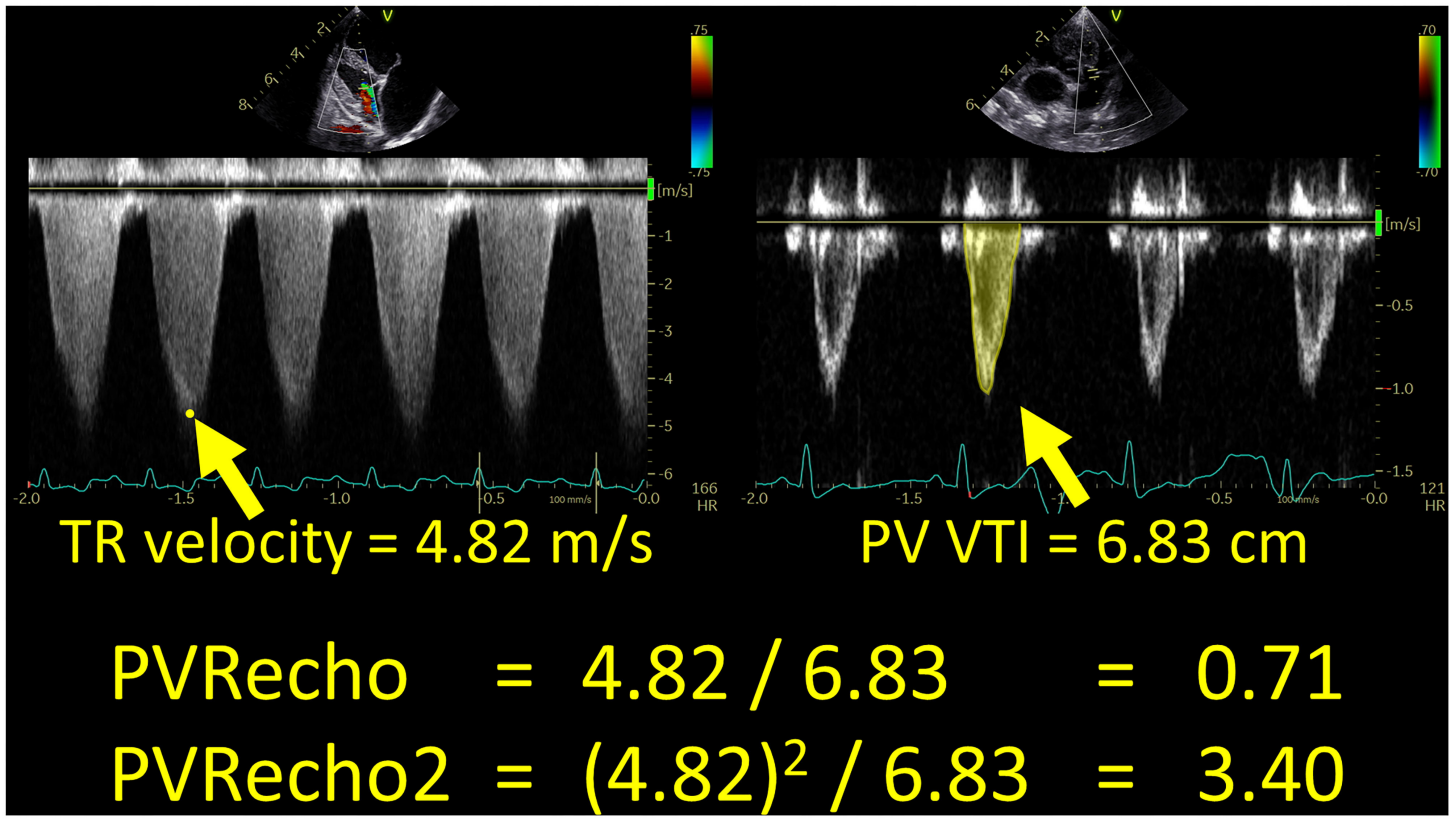
Echocardiographic estimation of pulmonary vascular resistance in a dog with high pulmonary hypertension probability. PV VTI, velocity–time integral of the pulmonary artery flow; PVRecho, pulmonary vascular resistance estimated by echocardiography; TR, tricuspid valve regurgitation.

The PVRecho measured by the two methods was calculated using the following formulas (11, 13) (Figure 1):


(6) $$\begin{array}{l} \text{PVRecho}=\frac{\text{peak TR velocity }\left(\text{m}/\text{s}\right)}{\text{PV VTI }\left(\text{cm}\right)} \end{array}$$

(7) $$\begin{array}{l} \text{PVRecho}2=\frac{{\left[\text{peak TR velocity }\left(\text{m}/\text{s}\right)\right]}^{2}}{\text{PV VTI }\left(\text{cm}\right)}\; \end{array}$$

**Figure 2:**
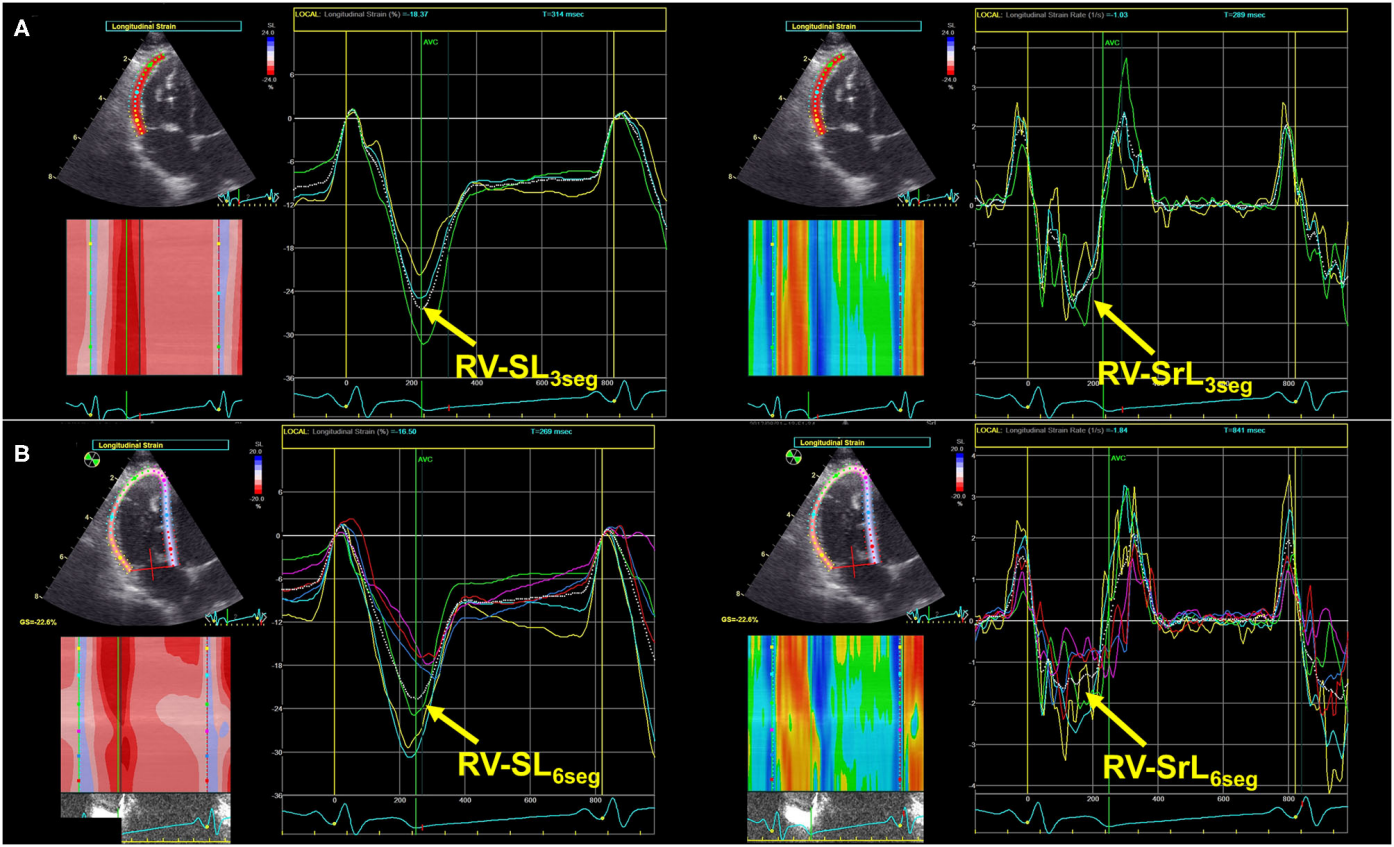
Right ventricular longitudinal strain (RV-SL) and strain rate (RV-SrL) assessed by two-dimensional speckle tracking echocardiography. **(A)** RV-SL and RV-SrL obtained from right ventricular free wall (RV-SL_3seg_ and RV-SrL_3seg_, respectively). **(B)** RV-SL and RV-SrL obtained from the global right ventricle (RV-SL_6seg_ and RV-SrL_6seg_, respectively).

As an indicator of intrinsic RV systolic function, we measured RV longitudinal strain and strain rate (RV-SL and RV-SrL, respectively) using the two-dimensional speckle tracking echocardiography (2D-STE) method. All 2D-STE analyses were performed using the same offline workstation as that used for standard echocardiography. RV-SL and RV-SrL were obtained from the RV focus view using the left ventricular four-chamber algorithm (16, 17, 27). The region of interest for 2D-STE was determined by manually tracing the RV endocardial border. Manual adjustments were made to include and track the entire myocardial thickness over the cardiac cycle when necessary. When the automated software could not track the myocardial regions, the regions of interest were retraced and recalculated. RV-SL and RV-SrL were measured using only RV free wall analysis (3seg), performed by tracing from the level of the lateral tricuspid annulus to the RV apex (Figure 2A), and RV global analysis (6seg), performed by tracing from the lateral tricuspid annulus to the septal tricuspid annulus *via* the RV apex (i.e., both RV free wall and interventricular septum) (Figure 2B). RV-SL was reported as the absolute value of the negative peak obtained from the strain wave (16, 17, 27). The RV-SrL was obtained from the strain rate wave and was reported as the absolute value of the negative peak during systole (27–29).

### Statistical Analysis

All statistical analyses were performed using commercially available software (R 2.8.1). Categorical data are expressed as absolute numbers and frequencies as percentages. Continuous data are reported as median (interquartile range).

Shapiro–Wilk test was performed to evaluate the normality of the data. Categorical indices were compared among the PH probability groups using Fisher's exact test. Continuous indices were compared among the PH probability groups using one-way analysis of variance with subsequent pairwise comparisons using Tukey's multiple comparison test for normally distributed data or the Kruskal–Wallis test with subsequent pairwise comparisons using the Steel–Dwass test for non-normally distributed data. All echocardiographic indices for right heart morphology and function were compared for the presence or absence of R-CHF using a Student's *t*-test for normally distributed data or the Mann–Whitney *U* test for non-normally distributed data. Additionally, univariate and multivariate logistic regression analyses were performed to evaluate the association between the presence of R-CHF and echocardiographic indices for the right heart, including PVRecho. After adjusting for multicollinearity, variables with *P* < 0.10 in the univariate analysis, were entered into the multivariate analysis. Results of logistic regression analyses were recorded as adjusted odds ratios and their respective 95% confidence intervals (CI). Receiver operating characteristic curves were created to calculate the area under the curve (AUC), sensitivity, and specificity, and to determine the optimal cutoff values required for evaluating the presence of R-CHF. The AUC was considered to have high accuracy if it was >0.9, moderate accuracy if it was 0.7–0.9, and low accuracy if it was 0.5–0.7 (30). The optimal cutoff value was defined as the value that minimized the distance between the curve and the upper left corner in the receiver operating characteristic curve.

Intra-observer variability measurements were performed by a single observer who performed all echocardiographic measurements (YY). The echocardiographic indices assessed in this study were obtained from nine dogs (three dogs in each PH probability group). All measurements were performed on two different days with >7-day intervals using the same cardiogram and cardiac cycles. A second blinded observer (HKa) measured the same indices to determine interobserver variability using the same echocardiogram and heart cycles. Variability of intra- and inter-observer measurements was quantified by the coefficient of variation (CV), which was calculated using the following formula:

$\text{CV }\left(\%\right)=\frac{\left(\text{standard deviation}\right)}{\left(\text{mean value}\right)}\times 100$

Intra- and inter-class correlation coefficients (ICCs) were also used to evaluate measurement variability. Low measurement variability was defined as CV < 10.0, and ICC > 0.70.

Statistical significance was set at *P* < 0.050 for all the analyses.

## Results

### Clinical Characteristics

Fifty-eight client-owned dogs with MMVD and TR were enrolled in this study. The dogs were of the following breeds: Chihuahua (*n* = 15, 22%), mixed breed (*n* = 7, 12%), Toy Poodle (*n* = 6, 10%), Shi Tzu (*n* = 5, 9%), Miniature Dachshund (*n* = 3, 5%), Maltese (*n* = 3, 5%), Miniature Schnauzer (*n* = 3, 5%), Papillon (*n* = 2, 3%), Pomeranian (*n* = 2, 3%), Cavalier King Charles spaniel (*n* = 2, 3%), Chinese Crested dog (*n* = 2, 3%), Norfolk terrier (*n* = 2, 3%), and one dog each from eight other breeds. Seventy-eight percent of dogs with MMVD received medical treatment from the referral hospital at the time of examination, which was either angiotensin converting enzyme inhibitors (low: *n* = 9; intermediate: *n* = 11; high: *n* = 20), pimobendan (low: *n* = 4; intermediate: *n* = 9; high: *n* = 14), sildenafil (low: *n* = 0; intermediate: *n* = 2; high: *n* = 5), loop diuretics (low: *n* = 0; intermediate: *n* = 0; high: *n* = 4), or a combination of these. The clinical characteristics and systemic blood pressure results are summarized in Table 1. Age, sex, body weight, and systemic arterial pressure showed no significant differences among the PH probability groups. The proportion of the ACVIM stage of MMVD and the presence of R-CHF was significantly associated with PH probability (*P* = 0.004 and *P* < 0.001, respectively).

**Table 1:** Clinical characteristics of the study population.

**Variables**	**Pulmonary hypertension probability**			***P***
	**Low**	**Intermediate**	**High**	
Number	16	19	23	
Age (year)	11 (9.6–13.5)	11.6 (9.8–13.2)	13.3 (12.2–14.6)	0.056
Sex (male/female)	10/6	13/6	11/13	0.451
Body weight (kg)	5.1 (3.3–7.6)	4.4 (3.3–6.9)	4.8 (2.5–7.2)	0.769
ACVIM Stage (B1/B2/C, D)	8/5/3	5/11/3	0/7/16	0.004
R-CHF (present/absent)	0/16	0/21	10/13	<0.001
Heart rate (bpm)	121 (109–152)	136 (120–151)	149 (127–158)	0.350
**Systemic arterial pressure**				
Systole (mmHg)	126 (110–146)	136 (118–148)	135 (106–150)	0.451
Mean (mmHg)	92 (81–105)	98 (83–107)	96 (88–112)	0.832

*Data are represented as median (interquartile range)*.

*ACVIM, American College of Veterinary Internal Medicine; R-CHF, right-sided congestive heart failure*.

### Echocardiographic Variables for the Right Heart

Table 2 shows the results of the echocardiographic variables, including the 2D-STE indices. Regarding the morphological indicators for the right heart, the RVIDd index, RVEDA index, and RVESA index were significantly higher in the high probability group than in the low and intermediate probability groups (RVIDd index: *P* = 0.006 [vs. low], *P* < 0.001 [vs. intermediate]; RVEDA index: *P* = 0.002 [vs. low], *P* < 0.001 [vs. intermediate]; RVESA index: *P* = 0.006 [vs. low], *P* = 0.002 [vs. intermediate]). Additionally, the PA/Ao ratio in the intermediate and high probability groups was significantly higher than that in the low probability group (*P* = 0.049 and *P* = 0.005, respectively).

**Table 2:** Results of echocardiographic indices in dogs with myxomatous mitral valve disease and pulmonary hypertension probability.

**Variables**	**Pulmonary hypertension probability**			***P***
	**Low**	**Intermediate**	**High**	
RVIDd index (mm/kg^0.327^)	6.3 (5.3–7.3)	5.8 (5.3–7.1)	7.7 (6.8–9.5)^*^^†^	<0.001
RVEDA index (cm^2^/kg^0.624^)	0.83 (0.73–0.96)	0.86 (0.75–0.96)	1.17 (1.00–1.33)^*^^†^	0.001
RVESA index (cm^2^/kg^0.628^)	0.43 (0.36–0.47)	0.43 (0.31–0.51)	0.57 (0.47–0.73)^*^^†^	0.003
PA/Ao	0.82 (0.76–0.86)	0.90 (0.83–0.97)^*^	0.93 (0.85–1.19)^*^	0.002
TAPSEn (cm/kg^0.284^)	7.2 (6.1–7.5)	7.5 (6.9–9.3)	8.0 (6.6–9.1)	0.205
RV FACn (%/kg^−0.097^)	54.9 (49.4–63.5)	61.1 (49.7–63.3)	57.6 (47.2–61.2)	0.551
RV s' (cm/s)	11.7 (9.6–12.8)	11.4 (9.5–12.8)	11.8 (8.6–16.1)	0.433
RV MPI	0.45 (0.32–0.62)	0.41 (0.33–0.53)	0.58 (0.40–0.69)	0.112
PV VTI (cm)	8.6 (6.8–10.7)	8.8 (6.9–10.6)	7.1 (5.3–8.3)^*^^†^	0.005
TR velocity (m/s)	2.7 (2.2–2.8)	3.2 (3.1–3.4)^*^	3.9 (3.5–5.0)^*^^†^	<0.001
PVRecho	0.27 (0.23–0.40)	0.35 (0.29–0.46)^*^	0.60 (0.48–0.80)^*^^†^	<0.001
PVRecho2	0.73 (0.51–1.08)	1.11 (0.99–1.38)^*^	2.46 (1.66–3.58)^*^^†^	<0.001
RV-SL_3seg_ (%)	29.3 (26.6–34.8)	31.0 (26.6–32.5)	25.1 (22.6–30.0)^†^	0.049
RV-SrL_3seg_ (%/s)	4.9 (3.6–6.9)	5.3 (3.8–8.4)	3.4 (2.8–6.1)	0.088
RV-SL_6seg_ (%)	25.8 (20.9–29.8)	27.4 (24.2–32.2)	20.6 (15.0–25.0)^*^^†^	0.003
RV-SrL_6seg_ (%/s)	3.7 (2.6–4.6)	3.9 (2.6–5.3)	2.9 (2.2–3.5)^†^	0.021

*Data are represented as median (interquartile range)*.

*3seg, only right ventricular free wall analysis; 6seg, right ventricular global analysis; PA/Ao, pulmonary artery to aortic diameter ratio; PVRecho, pulmonary vascular resistance estimated by echocardiography; PV VTI, velocity–time integral of the pulmonary artery flow; RV, right ventricular; RV FACn, RV fractional area change normalized by body weight; RV MPI, RV myocardial performance index; RV s', tissue Doppler imaging-derived peak systolic myocardial velocity of lateral tricuspid annulus; RVEDA index, end-diastolic RV area normalized by body weight; RVESA index, end-systolic RV area normalized by body weight; RVIDd index, end-diastolic RV internal dimension normalized by body weight; RV-SL, RV longitudinal strain; RV-SrL, RV longitudinal strain rate; TAPSEn, tricuspid annular plane systolic excursion normalized by body weight; TR, tricuspid valve regurgitation*.

* *Variables that was significantly different from the low probability group (P < 0.050)*.

† *Variables that was significantly different from the intermediate probability group (P < 0.050)*.

**Figure 3:**
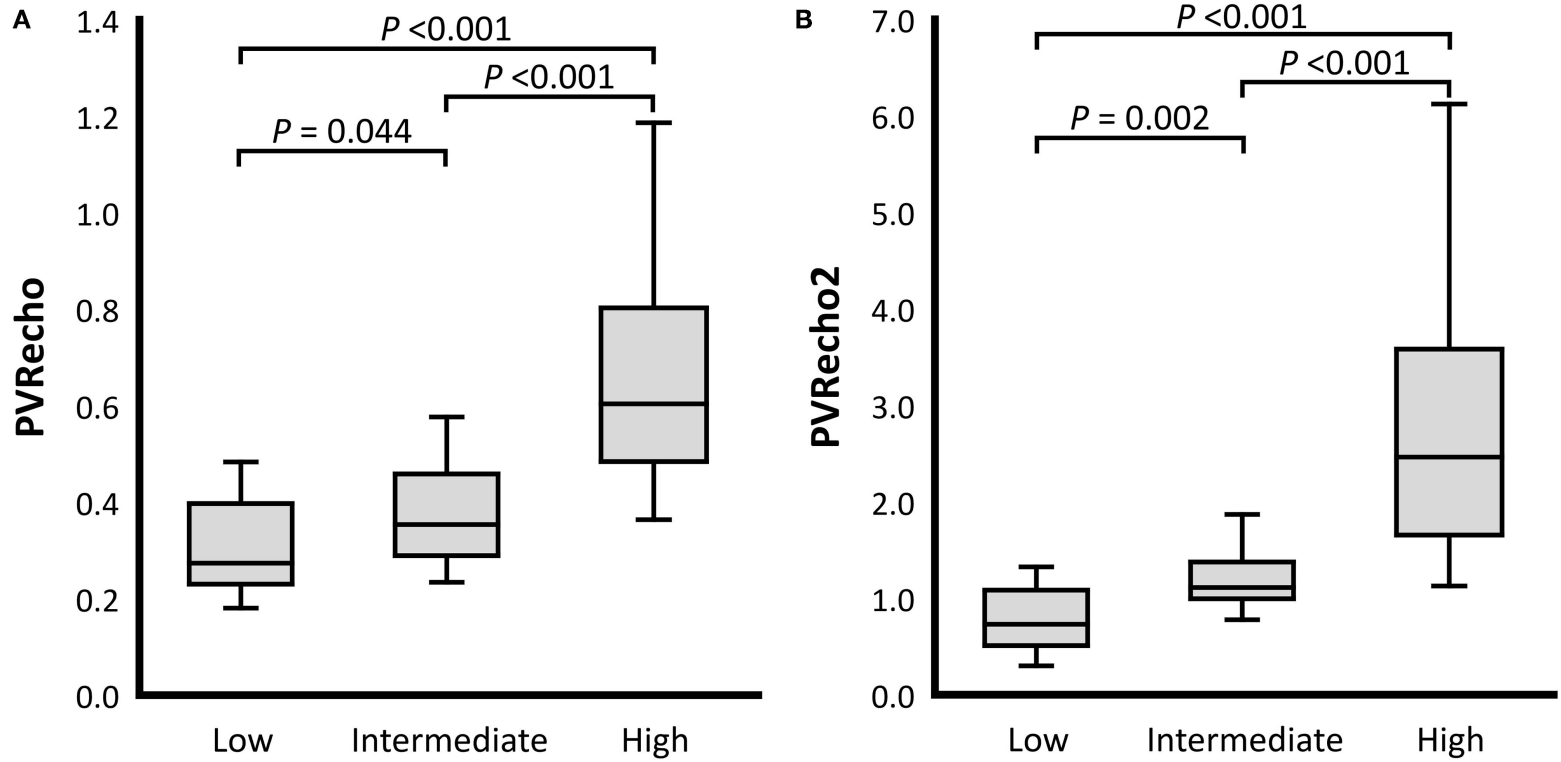
Box and whisker plots of the pulmonary vascular resistance estimated by echocardiography (PVRecho). The bottom of the box is 25%, the middle line is the median, the top of the box is 75%, and the whiskers represent the range. **(A)** PVRecho. **(B)** PVRecho2.

For the RV functional variables, TAPSEn, RV FACn, RV s', and RV MPI showed no significant differences among the PH probability groups (*P* = 0.205, *P* = 0.551, *P* = 0.433, and *P* = 0.112, respectively). On the other hand, PV VTI in the high probability group was significantly lower than that in the low and intermediate probability groups (PV VTI: *P* = 0.022 and *P* = 0.008, respectively). TR velocity was significantly different among all PH probability groups (*P* < 0.001). PVRecho and PVRecho2 were also significantly different among all PH probability groups (PVRecho: *P* = 0.044 [low vs. intermediate], *P* < 0.001 [low vs. high and intermediate vs. high]; PVRecho2: *P* = 0.002 [low vs. intermediate], *P* < 0.001 [low vs. high and intermediate vs. high]) (Figure 3). Regarding the 2D-STE indices, RV-SL_3seg_ in the high probability group was significantly lower than that in the intermediate probability group (*P* = 0.049). Additionally, RV-SL_6seg_ in the high probability group was significantly lower than that in the low and intermediate probability groups (*P* = 0.034 and *P* = 0.003, respectively). Although RV-SrL_3seg_ showed no significant difference among the PH probability groups, RV-SrL_6seg_ in the high probability group was significantly lower in the intermediate probability group (*P* = 0.023).

Table 3 summarizes the echocardiographic indices for the right heart compared between the presence and absence of R-CHF. The comparison among the PH probability groups, RVIDd index, RVEDA index, RVESA index, and PA/Ao were significantly higher in dogs with R-CHF. Additionally, certain RV functional indices, including RV MPI, PV VTI, TR velocity, PVRecho, PVRecho2, RV-SL_6seg_, and RV-SrL_6seg_, were significantly different between dogs with and without R-CHF. However, TAPSEn, RV FACn, RV s', RV-SL_3seg_, and RV-SrL_3seg_ showed no significant differences between dogs with and without R-CHF.

**Table 3:** Results of echocardiographic variables between the presence or absence of congestive heart failure.

**Variables**	**Right-sided congestive heart failure**		***P***
	**Present**	**Absent**	
RVIDd index (mm/kg^0.327^)	10.0 (8.3–12.0)	6.7 (5.4–7.4)	<0.001
RVEDA index (cm^2^/kg^0.624^)	1.36 (1.13–1.67)	0.86 (0.74–1.01)	0.003
RVESA index (cm^2^/kg^0.628^)	0.73 (0.61–0.88)	0.45 (0.35–0.52)	0.003
PA/Ao	1.00 (0.89–1.27)	0.87 (0.81–0.92)	0.006
TAPSEn (cm/kg^0.284^)	7.2 (6.0–8.5)	7.5 (6.7–8.3)	0.511
RV FACn (%/kg^−0.097^)	49.2 (36.9–63.1)	58.8 (49.8–62.4)	0.113
RV s' (cm/s)	11.8 (8.5–17.4)	11.4 (9.3–13.6)	0.308
RV MPI	0.65 (0.34–0.96)	0.44 (0.37–0.58)	0.046
PV VTI (cm)	5.9 (4.5–7.9)	8.4 (6.8–9.7)	0.002
TR velocity (m/s)	5.0 (4.3–5.1)	3.1 (2.8–3.5)	<0.001
PVRecho	0.76 (0.62–1.00)	0.38 (0.29–0.48)	<0.001
PVRecho2	3.32 (2.80–5.03)	1.16 (0.84–1.62)	<0.001
RV-SL_3seg_ (%)	25.5 (23.3–30.4)	28.8 (25.2–31.6)	0.395
RV-SrL_3seg_ (%/s)	4.4 (2.7–6.4)	4.9 (3.3–6.8)	0.452
RV-SL_6seg_ (%)	19 (13.3–24.3)	25.3 (20.9–28.4)	0.008
RV-SrL_6seg_ (%/s)	2.4 (1.8–3.6)	3.4 (2.6–4.6)	0.017

*Data are represented as median (interquartile range)*.

*3seg, only right ventricular free wall analysis; 6seg, right ventricular global analysis; PA/Ao, pulmonary artery to aortic diameter ratio; PVRecho, pulmonary vascular resistance estimated by echocardiography; PV VTI, velocity–time integral of the pulmonary artery flow; RV, right ventricular; RV FACn, RV fractional area change normalized by body weight; RV MPI, RV myocardial performance index; RV s', tissue Doppler imaging-derived peak systolic myocardial velocity of lateral tricuspid annulus; RVEDA index, end-diastolic RV area normalized by body weight; RVESA index, end-systolic RV area normalized by body weight; RVIDd index, end-diastolic RV internal dimension normalized by body weight; RV-SL, RV longitudinal strain; RV-SrL, RV longitudinal strain rate; TAPSEn, tricuspid annular plane systolic excursion normalized by body weight; TR, tricuspid valve regurgitation*.

The intra- and inter-observer measurement variability results are summarized in Table 4. For inter-observer variability, all variables assessed in this study showed low measurement variability. However, RV FAC and RV MPI did not meet the criteria of low measurement variability based on CV < 10.0, and ICC > 0.70.

**Table 4:** Intra- and inter-observer measurement variability in echocardiographic variables assessed in this study.

**Variables**	**Intra-observer**			**Inter-observer**		
	**CV (%)**	**ICC**	***P***	**CV (%)**	**ICC**	***P***
RVIDd	3.2	0.97	<0.001	7.6	0.95	<0.001
RVEDA	4.0	0.99	<0.001	6.4	0.95	<0.001
RVESA	5.6	0.94	<0.001	7.6	0.96	0.001
PA/Ao	7.1	0.89	0.002	8.2	0.86	0.001
RV FAC	5.2	0.91	<0.001	7.5	0.67	0.001
TAPSE	3.5	0.90	<0.001	6.4	0.80	0.012
RV s'	2.7	0.97	<0.001	3.8	0.95	<0.001
RV MPI	9.1	0.86	0.002	12.5	0.72	0.044
PV VTI	3.8	0.98	<0.001	5.5	0.94	<0.001
TR velocity	0.9	0.99	<0.001	0.9	0.99	<0.001
PVRecho	3.7	0.99	<0.001	5.4	0.96	<0.001
PVRecho2	3.6	0.99	<0.001	5.4	0.97	<0.001
RV-SL_3seg_	4.5	0.93	<0.001	5.2	0.92	<0.001
RV-SrL_3seg_	6.4	0.95	<0.001	9.7	0.89	<0.001
RV-SL_6seg_	5.6	0.91	<0.001	6.9	0.86	<0.001
RV-SrL_6seg_	6.1	0.94	<0.001	8.0	0.93	<0.001

*3seg, only RV free wall analysis; 6seg, RV global analysis; CV, coefficient of variation; ICC, intra- or inter-class correlation coefficients; PA/Ao, pulmonary artery to aortic diameter ratio; PVRecho, pulmonary vascular resistance estimated by echocardiography; PV VTI, velocity–time integral of the pulmonary artery flow; RV, right ventricular; RV FAC, RV fractional area change; RV MPI, RV myocardial performance index; RV s', tissue Doppler imaging-derived peak systolic myocardial velocity of lateral tricuspid annulus; RVEDA, end-diastolic RV area; RVESA, end-systolic RV area; RV-SL, RV longitudinal strain; RV-SrL, RV longitudinal strain rate; TAPSE, tricuspid annular plane systolic excursion; TR, tricuspid valve regurgitation*.

### Logistic Regression Analysis

In the univariate analyses that evaluated the association between echocardiographic variables for the right heart and the presence of R-CHF, an association was observed between the presence of R-CHF and increased RVIDd index, RVEDA index, RVESA index, PA/Ao, RV MPI, TR velocity, PVRecho, and PVRecho2, and decreased RV FACn, PV VTI, RV-SL_6seg_, and RV-SrL_6seg_ (Table 5). After adjusting for confounding factors, five indices, including RVIDd index, PA/Ao, RV MPI, PVRecho2, and RV-SL_6seg_, were included in the multivariate model, and the RVIDd index and PVRecho2 remained significant in the multivariate analysis.

**Table 5:** Significant variables in logistic regression analysis to evaluate the association between right-sided congestive heart failure and echocardiographic variables.

**Variables**	**Univariate analysis**		**Multivariate analysis**	
	**Non-adjusted odds ratio (95% CI)**	***P***	**Adjusted odds ratio (95% CI)**	***P***
RVIDd index (mm/kg^0.327^)	5.4 (1.7–16.5)	<0.001	3.2 (1.2–12.2)	0.036
RVEDA index (cm^2^/kg^0.624^)	2.1 (1.3–3.4)	<0.001		
RVESA index (cm^2^/kg^0.628^)	4.1 (1.8–9.5)	<0.001		
PA/Ao (0.1)	1.8 (1.2–2.6)	0.005		
RV FACn (%/kg^−0.097^)	1.1 (1.0–1.2)	0.029		
RV MPI (0.1)	1.5 (1.1–2.0)	0.019		
PV VTI (cm)	2.0 (1.2–3.3)	0.006		
TR velocity (m/s)	1.3 (1.2–1.6)	<0.001		
PVRecho (0.1)	4.5 (1.7–12.1)	0.003		
PVRecho2 (0.1)	1.3 (1.1–1.5)	0.001	1.2 (1.1–1.4)	0.026
RV-SL_6seg_ (%)	1.2 (1.0–1.3)	0.014		
RV-SrL_6seg_ (0.1 %/s)	1.1 (1.0–1.2)	0.018		

*6seg, right ventricular global analysis; PA/Ao, pulmonary artery to aortic diameter ratio; PVRecho, pulmonary vascular resistance estimated by echocardiography; PV VTI, velocity–time integral of the pulmonary artery flow; RV, right ventricular; RV FACn, RV fractional area change normalized by body weight; RV MPI, RV myocardial performance index; RVEDA index, end-diastolic RV area normalized by body weight; RVESA index, end-systolic RV area normalized by body weight; RVIDd index, end-diastolic RV internal dimension normalized by body weight; RV-SL, RV longitudinal strain; RV-SrL, RV longitudinal strain rate; TR, tricuspid valve regurgitation*.

The results of the receiver operating characteristic curves of the significant variables in the univariate analysis are summarized in Table 6. The RVIDd index, RVESA index, TR velocity, PVRecho, and PVRecho2 had a high accuracy in detecting the presence of R-CHF, and the RVEDA index, PA/Ao, PV VTI, RV-SL_6seg_, and RV-SrL_6seg_ had moderate accuracy, and the other variables had low accuracy.

**Table 6:** Area under the curve and optimal cutoff of the significant variables in the univariate logistic regression analyses to detect the presence of right-sided congestive heart failure.

**Variables**	**AUC (95% CI)**	**Cutoff**	**Sensitivity**	**Specificity**
RVIDd index (mm/kg^0.327^)	0.952 (0.887–1.000)	7.68	0.90	0.88
RVEDA index (cm^2^/kg^0.624^)	0.886 (0.726–1.000)	1.12	0.88	0.90
RVESA index (cm^2^/kg^0.628^)	0.902 (0.760–1.000)	0.54	0.82	0.90
PA/Ao (0.1)	0.776 (0.598–0.954)	0.96	0.70	0.82
RV FACn (%/kg^−0.097^)	0.684 (0.443–0.925)	51.3	0.70	0.74
RV MPI (0.1)	0.694 (0.470–0.917)	0.57	0.70	0.73
PV VTI (cm)	0.802 (0.657–0.947)	7.0	0.70	0.70
TR velocity (m/s)	0.907 (0.850–1.000)	4.52	0.80	0.96
PVRecho (0.1)	0.964 (0.921–1.000)	0.60	0.90	0.94
PVRecho2 (0.1)	0.974 (0.939–1.000)	2.46	0.90	0.94
RV-SL_6seg_ (%)	0.748 (0.585–0.911)	22.40	0.70	0.72
RV-SrL_6seg_ (0.1 %/s)	0.754 (0.582–0.926)	2.55	0.70	0.78

*6seg, right ventricular global analysis; AUC, area under the curve; PA/Ao, pulmonary artery to aortic diameter ratio; PVRecho, pulmonary vascular resistance estimated by echocardiography; PV VTI, velocity–time integral of the pulmonary artery flow; RV, right ventricular; RV FACn, RV fractional area change normalized by body weight; RV MPI, RV myocardial performance index; RVEDA index, end-diastolic RV area normalized by body weight; RVESA index, end-systolic RV area normalized by body weight; RVIDd index, end-diastolic RV internal dimension normalized by body weight; RV-SL, RV longitudinal strain; RV-SrL, RV longitudinal strain rate; TR, tricuspid valve regurgitation*.

*Optimal cutoff was defined as that which minimized the distance between the curve and the upper left corner in the ROC curve*.

## Discussion

This study was the first to evaluate the clinical utility of PVRecho in dogs with MMVD, classified according to the PH probability. Several echocardiographic variables such as TAPSEn, RV FACn, RV s', and RV MPI, showed no statistical significance among the PH probability groups; RV-SL_3seg_, RV-SL_6seg_, PV VTI, TR velocity, PVRecho, and PVRecho2 showed significant worsening with the increase in PH probability. Additionally, logistic regression analysis revealed a significant association between the presence of R-CHF and increased RVIDd index and PVRecho2. Our results suggest that PVR estimated by echocardiography may provide additional information for the diagnosis and stratification of PH in dogs with MMVD, reflecting the increase in PVR and associated RV adaptation and/or the progression to Cpc-PH.

In this study, PVRecho measured by the two methods was significantly higher as the PH probability increased. In particular, those in the high probability group showed substantially higher values compared to those in the low and intermediate probability groups. These results suggest that dogs with high PH probability might have an increase in PVR in addition to the increase in PAP. Furthermore, some dogs with high PH probability may have pulmonary arterial remodeling (i.e., the development of Cpc-PH). Higher PVRecho observed in this study may reflect increased PVR attributable to the significant pulmonary arterial remodeling and detect the Cpc-PH condition. However, since not all dogs have undergone histopathological examination, further studies that compare echocardiographic and histopathological findings are warranted in the future. Additionally, the PVR estimated by echocardiography was calculated with PV VTI, which reflects RV performance and TR velocity, which indicates RV afterload. Therefore, these indices might also reflect RV adaptation associated with increased PVR. Although TR velocity showed the same tendency as PVRecho, a previous study has reported that systolic PAP estimated by TR velocity showed poor agreement with that measured by catheterization (31), and ACVIM consensus stated that PH diagnosis by TR velocity alone should be avoided (1). Furthermore, our previous study reported that RV compensated for mild pressure overload by hyperactivation and decompensated for moderate-to-severe and chronic pressure overload in dogs with experimentally induced PH (27), suggesting that the pseudo-normalization of RV systolic function cannot be avoided as PH progresses. Therefore, the overall assessment of RV performance and afterload, such as PVRecho and PVRecho2, might be more useful for the clinical assessment of PH rather than the assessment using TR velocity and/or RV performance variables alone.

Logistic regression analysis in this study revealed a significant association between the presence of R-CHF and increased PVRecho2 with high AUC, sensitivity, and specificity. PVRecho was considered an independent factor for mortality in human patients with interstitial lung disease (12). Additionally, the development of Cpc-PH has been reported to be associated with RV failure and poor prognosis in patients with post-capillary PH (5, 32). Our results also suggest that increased PVR estimated by echocardiography may be a poor prognostic factor in dogs with MMVD, reflecting the increased PVR and associated RV maladaptation and/or the progression to Cpc-PH. In this study, an increased RVIDd index was also associated with the presence of R-CHF with high AUC, sensitivity, and specificity, as well as PVRecho2. We previously described RV maladaptation against RV afterload in dogs with chronic PH and that RV dysfunction would induce RV dilatation to maintain RV cardiac output (27). Dogs with R-CHF in this study also showed RV dysfunction based on a decrease in RV-SL and RV-SrL and increased RV afterload based on TR velocity. Additionally, previous studies have reported that right heart dilatation is associated with the presence of R-CHF and shorter survival time (19, 33). Therefore, our results suggest that the PVRecho2 and RVIDd index may provide additional information to determine the presence of R-CHF in dogs with MMVD.

In our study population, PVRecho and PVRecho2 showed equivalent power for the stratification of PH probability and the detection of the presence of R-CHF. A previous human study reported that PVRecho2 was more reliable in estimating the catheterization-derived PVR than PVRecho in patients with substantially elevated PVR (11). Although not all dogs underwent right heart catheterization, our study results suggest that PVRecho calculated by both methods might be clinically useful for the diagnosis and stratification of PH in dogs with MMVD. However, the small study population of dogs with progressive PH and R-CHF might have affected the results in this study. Unfortunately, only a few dogs with post-capillary PH might progress to Cpc-PH. Further studies that accumulate more dogs with severe PH are expected in the future.

This study has several limitations. First, since not all dogs had undergone right heart catheterization, the definitive diagnosis of PH and the actual PVR value could not be completely clarified. Additionally, because not all dogs have undergone complete differential diagnosis, diseases that might increase PAP and/or PVR other than MMVD (i.e., pre-capillary PH) could not be completely ruled out (1). Second, few cases of misdiagnosis may have occurred in some dogs with R-CHF. Since none of the dogs underwent complete abdominal ultrasonography, we may not have identified some dogs with mild ascites. Third, some medications, especially pimobendan, sildenafil, and loop diuretics, might have affected RV function and hemodynamics. Especially, sildenafil, which would decrease PVR, might also affect our results of PVRecho. Finally, because the number of dogs with R-CHF in our study population was relatively small, the possibility of selection bias and insufficient power to detect statistical differences could not be excluded.

In conclusion, PVR estimated by echocardiography showed a significantly high value as the PH probability increased. These results suggest that these novel non-invasive values might be useful tools for the diagnosis and stratification of PH in dogs with MMVD. Additionally, multivariate logistic regression analysis revealed a significant association between the presence of R-CHF and increased PVRecho2 and the RVIDd index. Further studies that increase the study population and compare right heart catheterization- and echocardiography-derived PVR are warranted to validate the accuracy and utility of PVR estimated by echocardiography in the future.

## Data Availability Statement

The raw data supporting the conclusions of this article will be made available by the authors, without undue reservation.

## Ethics Statement

The animal study was reviewed and approved by Ethical Committee for Animal Use of the Nippon Veterinary and Life Science University Veterinary Medical Teaching Hospital. Written informed consent was obtained from the owners for the participation of their animals in this study.

## Author Contributions

RS provided the academic direction, performed the concept/design, data interpretation, critical revision of the manuscript, and approved the manuscript. YY performed the concept/design, data analysis/interpretation, drafting the manuscript, and critical revision of the manuscript. HKa performed the data analysis as a second observer. TS, TT, HM, and HKo performed data interpretation, critically revised the manuscript, and approved the manuscript. All authors contributed to the article and approved the submitted version.

## Funding

This work was partially supported by the Japan Society for the Promotion of Science (JSPS) KAKENHI Grant Number 20K15667.

## Conflict of Interest

The authors declare that the research was conducted in the absence of any commercial or financial relationships that could be construed as a potential conflict of interest.

## Publisher's Note

All claims expressed in this article are solely those of the authors and do not necessarily represent those of their affiliated organizations, or those of the publisher, the editors and the reviewers. Any product that may be evaluated in this article, or claim that may be made by its manufacturer, is not guaranteed or endorsed by the publisher.

## Abbreviations

2D-STE: two-dimensional speckle tracking echocardiography
3seg: only right ventricular free wall analysis
6seg: right ventricular global analysis
CI: confidence interval
CV: coefficient of variation
ICC: intra- or inter-class correlation coefficients
MMVD: myxomatous mitral valve disease
PA/Ao: pulmonary artery to aortic diameter ratio
PAP: pulmonary arterial pressure
PH: pulmonary hypertension
PVR: pulmonary vascular resistance
PVRecho: pulmonary vascular resistance estimated by echocardiography
PV VTI: velocity-time integral of the pulmonary artery flow
RV: right ventricular
RV FACn: right ventricular fractional area change normalized by body weight
RV MPI: right ventricular myocardial performance index
RV s': tissue Doppler imaging-derived peak systolic myocardial velocity of lateral tricuspid annulus
RVEDA index: end-diastolic right ventricular area normalized by body weight
RVESA index: end-systolic right ventricular area normalized by body weight
RVIDd index: end-diastolic right ventricular internal dimension normalized by body weight
RV-SL: right ventricular longitudinal strain
RV-SrL: right ventricular longitudinal strain rate
TAPSEn: tricuspid annular plane systolic excursion normalized by body weight
TR: tricuspid valve regurgitation.
